# Validation of key behaviourally based mental health diagnoses in administrative data: suicide attempt, alcohol abuse, illicit drug abuse and tobacco use

**DOI:** 10.1186/1472-6963-12-18

**Published:** 2012-01-23

**Authors:** Hyungjin Myra Kim, Eric G Smith, Claire M Stano, Dara Ganoczy, Kara Zivin, Heather Walters, Marcia Valenstein

**Affiliations:** 1 Center for Statistical Consultation and Research, 3555 Rackham, University of Michigan, Ann Arbor, MI 48109-1070, USA; 2 Department of Veterans Affairs, Ann Arbor Center of Excellence (COE), Serious Mental Illness Treatment Resource and Evaluation Center (SMITREC), Ann Arbor, MI, 48105, USA; 3 Center for Health Quality, Outcomes and Economic Research, Edith Nourse Rogers Memorial VA Hospital, 200 Springs Road, Bedford, MA 01730, USA; 4 Department of Psychiatry, 55 Lake Avenue, University of Massachusetts Medical School, Worcester, MA 01655, USA; 5 Department of Psychiatry, 4250 Plymouth Rd., University of Michigan Medical School, Ann Arbor, MI, 48109-5765, USA

## Abstract

**Background:**

Observational research frequently uses administrative codes for mental health or substance use diagnoses and for important behaviours such as suicide attempts. We sought to validate codes (*International Classification of Diseases, 9^th ^edition, clinical modification *diagnostic and E-codes) entered in Veterans Health Administration administrative data for patients with depression versus a gold standard of electronic medical record text ("chart notation").

**Methods:**

Three random samples of patients were selected, each stratified by geographic region, gender, and year of cohort entry, from a VHA depression treatment cohort from April 1, 1999 to September 30, 2004. The first sample was selected from patients who died by suicide, the second from patients who remained alive on the date of death of suicide cases, and the third from patients with a new start of a commonly used antidepressant medication. Four variables were assessed using administrative codes in the year prior to the index date: suicide attempt, alcohol abuse/dependence, drug abuse/dependence and tobacco use.

**Results:**

Specificity was high (≥ 90%) for all four administrative codes, regardless of the sample. Sensitivity was ≤75% and was particularly low for suicide attempt (≤ 17%). Positive predictive values for alcohol dependence/abuse and tobacco use were high, but barely better than flipping a coin for illicit drug abuse/dependence. Sensitivity differed across the three samples, but was highest in the suicide death sample.

**Conclusions:**

Administrative data-based diagnoses among VHA records have high specificity, but low sensitivity. The accuracy level varies by different diagnosis and by different patient subgroup.

## Background

In many administrative data-based studies, most variables, including primary outcomes, are based on the *International Classification of Diseases (ICD), clinical modification *codes (ICD-9 or ICD-10). Although the criteria for these diagnoses codes are clearly delineated, the primary purpose of these codes in many health systems is for billing. However, these codes are often used in health services research.

Administrative codes may have issues with sensitivity, specificity or accuracy when used for research purposes. Some level of financial incentive exists for the clinicians and billing clerks to note all relevant diagnoses (e.g., slightly higher billing revenues for the clinician or the system). However, even if each diagnosis or condition of interest covered in an encounter is faithfully entered, other diagnoses that were not a clinical focus in a particular encounter will not be entered. In addition, a desire to avoid stigma may play a role in the under-coding of behavioural health issues, such as substance dependence or suicide attempts. As a further complication, the diagnostic criteria for some of these behaviourally-based conditions are more subjective than for medical conditions, which may interplay with desire to avoid stigma in using certain diagnostic codes. For instance, dependence criteria require clinicians to make a judgment about whether behaviours "substantially impair" the patient. Similarly, suicide attempt diagnoses often call for a judgment of whether the patient intended to harm oneself. Lastly, some codes, such as the E-codes used for suicide attempts, generate no financial reimbursement, and the only incentive for the clinician to enter this information is typically to better inform future care of the patient.

As a result, it has long been recognized that administrative codes sub-optimally represent a patient's condition and the totality of all their comorbid illnesses, especially for conditions relating to mental illness [1]. Validation of such coding with individual chart review is desirable [2] because large health care organizations such as the Veterans Affairs (VA) Health Systems, Veterans Health Administration (VHA), use these administrative data for quality improvement purposes, to assess patient outcomes, and to determine health services utilization [3,4]. With increasing numbers of outcomes and health services research studies based exclusively or primarily on administrative data, knowledge of the accuracy of various potential research variables typically obtained from administrative data is highly desirable, as the validity of any conclusions will depend largely on the validity of such data.

VHA and non-VHA researchers have compared medical charts and administrative records, and studies have reported that the quality of VHA data is steadily improving [3]. Szeto *et al*. found the sensitivities and specificities for several medical diagnoses in the VHA administrative data to be high with sensitivity greater than 80% for 8 of 9 diagnoses that are relevant to the choices of hypertension medication and higher than 91% specificity for all 9 diagnoses [5]. Studies that have validated administrative data for mental health services research have focused on diagnoses such as schizophrenia or depression [6,7], a range of mental health service provision by primary care physicians [8,9], or performance measures [10-12]. A study by Kashner *et al*. comparing medical charts and administrative records of inpatient VHA discharges in 1995 found 93.7% agreement for alcohol dependence syndrome and 95.2% for drug dependence [13]. More recently, a Canadian study reported low sensitivity and high specificity for alcohol abuse and for drug abuse by comparing ICD-9 based diagnoses against the chart diagnoses in patients admitted in 2003 at four teaching hospitals in Alberta [14]. However, no study, to our knowledge has validated behavioural mental health variables in patients with depression.

VHA patients are different from the general population in that they have higher rates of mental illness and substance abuse, and patients with depression have higher rates of co-morbid substance abuse compared to those without mental illness. This study examines discrepancies between administrative data and chart notes with regard to behaviourally based mental health diagnoses in a VHA population with depression. Specifically, we sought to assess the validity of diagnoses based on ICD codes and E-codes for four variables frequently used in administrative data-based mental health studies: suicide attempt, alcohol abuse or dependence, drug abuse or dependence, and tobacco use disorder. The validation was carried out by comparing diagnostic coding and individual chart review data, using the Veterans Health Administration's comprehensive [electronic medical record] computerized patient record system. Though chart notation is not expected to be fully comprehensive or without errors, we expected it to be more comprehensive than the diagnostic coding.

Given the complexity of factors likely to influence administrative coding of these conditions, we did not hypothesize which of the administrative codes may have the highest levels of sensitivity or specificity for data recorded in charts. However, given low numbers of administrative codes for suicide attempt, we hypothesized that suicide attempt codes may have low sensitivity for attempts noted in the medical chart notations.

## Methods

This retroactive chart abstraction study was conducted as a nested case control study as part of a larger pharmacoepidemiologic study to compare suicide risks across different antidepressants. The study included three random samples made of 368 patients who died by suicide, 362 control patients, and 571 new users of antidepressants. The samples were selected from the cohort of Veterans Health Administration (VHA) patients identified using administrative data as being in VHA depression treatment, with either two diagnoses of a depressive disorder or a depression diagnosis and an antidepressant start between 4/1/1999 and 9/30/2004 [4]. The study was conducted with institutional review board approval from the Veterans Affairs Health System.

The *suicide death sample *was randomly selected from individuals who died of suicide, stratified by year of entry into the depression cohort, four geographic regions of the patient's VHA facility of most use and gender. Suicide deaths were determined using data from the National Death Index (NDI), which is considered the "gold standard" in US mortality databases [15]. The sampling fraction was proportional to that of the total suicide population, except females were over-sampled (15% females to 85% males) within each stratum due to small number of females in the VHA who completed suicides. The *control sample *was obtained by selecting a random patient to match each patient in the suicide death sample on age (+/- 5 years) from those in the same stratum as the case patient and alive on the date of suicide (i.e., index date). This last step was done in order to assign an index date to determine diagnoses for control sample patients. The *antidepressant new user sample *was selected from the depression cohort, from the subgroup of those newly starting one of the seven most commonly used antidepressants: bupropion, citalopram, fluoxetine, mirtazapine, paroxetine, sertraline and venlafaxine. A new start of an antidepressant was defined as a start of an antidepressant after no antidepressant fills for at least 6 months. These seven antidepressants make up more than 90% of all new antidepressant fills. Again a random sample was selected, stratified by region, year of new start and the seven antidepressant agents with approximately equal number of patients across strata (i.e., disproportionate sampling was used).

### Index Date

The index date was defined as date of suicide death for patients in the suicide death sample and also for patients in the control sample (who were alive on that date). For the new antidepressant user group, the index date was the date of the new antidepressant start. Behavioural variables of interest were assessed using all administrative and chart note data for the year prior to and including the index dates. We chose to assess behavioural variables during the entire one year period because in research studies comorbid health conditions are not typically determined from a single encounter but based on all encounters from a longer period [4].

### Administrative Data Diagnoses

Administrative data variables were based on diagnoses recorded in any diagnosis field of inpatient stays and outpatient visits. Diagnoses were based on the *International Classification of Diseases, Ninth Revision, clinical modification *(*ICD-9*) diagnoses codes. Alcohol and drug indicators included diagnoses of current alcohol or drug abuse and/or dependence. If the ICD-9 diagnoses code indicated the alcohol or drug disorder was in remission, abuse or dependence was considered not present at that visit. However, if a diagnosis of substance dependence/abuse was recorded at any encounter during the year, the patient was identified as having a substance dependence/abuse disorder, even if a remission code was recorded later in the year. Additional file 1 shows the included and excluded diagnoses for each of the four key behavioural variables. Drug dependence/abuse diagnoses included cocaine, opioids, cannabis, barbiturates, amphetamines, hallucinogens and other specified or unspecified drugs.

### Chart Abstracted Diagnoses

Charts were abstracted by four trained reviewers. Manual chart reviews were completed with the aid of a previously validated electronic medical record search engine (EMERSE), which highlights words in pre-defined search bundles [16]. Search bundles were developed, pilot-tested and refined for each variable and made to contain terms that would broadly capture all notations related to the specific conditions (i.e., "suicidal ideation" or "hurt" for the suicidal attempt variable.) Four chart reviewers underwent training with pilot data to resolve discrepancies and to improve accuracy and agreement in abstraction. However, for abstractions included in this study, 92% of study patients were reviewed by one reviewer with the aid of EMERSE. The reviewers were blinded to administrative data and the sample to which each patient belonged.

For *suicide attempt*, any notation regarding an attempt at any time during one year prior to the index date such as "Client was brought to ER after wife found him with wrists slashed" was considered as presence of suicide attempt. For alcohol use, *problem use, abuse or dependence *was considered present if, for example, the number of drinks per session was noted to be on or above the binge drinking threshold (4 drinks per session for women and 5 drinks per session for men), the clinician instructed patients to stop or reduce their drinking, or referred them to a substance use treatment program. Alcohol problem use/abuse/dependence was not considered present in chart notes if alcohol use was not mentioned, no use was reported or use was reported without problem. For other drug use, *illicit drug problem use/abuse/dependence *included any illicit substance use, other than marijuana. For patients who reported marijuana use only, problem use included those who reported problem behaviours or were instructed to stop use by their clinician. *Tobacco use *was considered present if any notation of current smoking by the patient was recorded during the year prior to the index date, either as part of the mandatory VHA yearly tobacco screening or elsewhere in the record.

### Data Analysis

Within each patient sample, percent agreement and kappa values were calculated to assess agreement in the four behavioural variables determined by administrative data versus chart abstraction. All measures of accuracy, including sensitivity, specificity, positive predictive value (PPV) and negative predictive value (NPV), were calculated using chart notation as the "gold standard." In addition to accuracy measures for each sample, we also calculated unbiased estimates of various accuracy measures for the entire depression cohort during the study period from 4/1/1999 to 9/30/2004. This was done using the combined mutually exclusive samples of suicide deaths and controls where the estimates were adjusted for sampling weights with each observation weighted inversely by the number of people each represents in the full depression cohort based on the sampling strata. Weighted accuracy estimates based on the antidepressant user sample were also calculated as unbiased accuracy estimates of a cohort of patients newly starting an antidepressant during the study period. We also did the analyses by age groups for alcohol problem drinking/abuse/dependence, our most common set of diagnoses. All analyses were done using Stata 10.1 (StataCorp LP, College Station, TX).

## Results

Table 1 shows demographic clinical utilization characteristics during the one year prior to the index date by the different samples. Overall, the samples were 76% white, 88% male and 4% Hispanic. On average, 85% of patients had more than one visit in the 12 months prior to the index date from which to abstract data, with a slightly lower percentage having more than one visit in the antidepressant new user sample.

**Table 1:** Patient characteristics by the three different samples

	*Suicide Deaths*	*Controls*	*AD New Users*
	***(N = 368)***	***(N = 362)***	***(N = 571)***

***Characteristics***	***N (%)***	***N (%)***	***N (%)***

Male	312 (84.8)	311 (85.9)	520 (91.1)

Hispanic	8 (2.2)	17 (4.7)	26 (4.6)

White	290 (78.8)	274 (75.7)	431 (75.5)

Age (mean (SD))	57.5 (14.3)	57.6 (14.5)	56.2 (14.2)

0 Outpatient Visit^a^	31 (8.4)	34 (9.4)	15 (2.6)

1 Outpatient Visit^a^	19 (5.2)	12 (3.3)	80 (14.0)

> 1 Outpatient Visit^a^	318 (86.4)	316 (87.3)	476 (83.4)

Abbreviation: AD is antidepressants.

^a ^During the year of assessment considered for this study.

### Suicide Attempt

Table 2 shows the various measures by the different samples. Percent agreement for suicide attempt was lowest (87.8%) in the sample of patients who eventually died by suicide, but higher in the control sample (99.2%) and in the new user sample (97.0%). Sensitivity of administrative codes for suicide attempt was low across all samples, with highest sensitivity of 17.0% in the suicide death sample. Specificity was 100% for both the control sample and the antidepressant new user sample and was 99.7% for the suicide death sample. In the suicide death sample, one patient had an attempt coded in administrative data on the date of completed suicide, but without a chart notation. Sampling fraction-adjusted (weighted) sensitivity for suicide attempt was 2.1% and the specificity 99.9% for the depression cohort, i.e., the combined suicide death and control samples.

**Table 2:** Suicide attempt: comparison of administrative data using E-codes against suicide attempt notation in chart**^c^**, both during the past 12 months prior to and including the index date

	***Unweighted Estimates, %***^b ^***(N in fraction)***				
***Samples***	***Agreement ***	***Sensitivity***	***Specificity***	***PPV***	***NPV***

Suicide Death	88 (323/368)	17 (9/53)	100 (314/315)	90 (9/10)	88 (314/358)

Control	99 (359/362)	0 (0/3)	100 (359/359)	NA1	99 (359/362)

AD New User	97 (554/571)	11 (2/19)	100 (552/552)	100 (2/2)	97 (552/569)

Combined**^a^**	93 (682/730)	16 (9/56)	100 (673/674)	90 (9/10)	94 (673/720)

	***Weighted Estimates, %***^b ^***(95% confidence interval)***				

Suicide Death	87 (82, 92)	19 (5, 32)	100 (100, 100)	96 (85, 100)	87 (82, 91)

Control	100 (99, 100)	0	100	NA1	100 (99, 1.00)

AD New User	98 (95, 100)	25 (0, 72)	100	NA2	98 (95, 100)

Combined^a^	100 (99, 100)	2 (0, 5)	100 (100, 100)	NA1	100 (99, 100)

Abbreviation: NA1 is not appropriate (inappropriate to estimate because the N of the denominator is 0 for the unweighted estimate and thus the corresponding weighted estimates based partly or entirely on the control sample are also inappropriate); NA2 is not appropriate (inappropriate because weighting would be based on N = 2); AD is antidepressant; PPV is positive predictive value of an administrative diagnosis; NPV is the negative predictive value of an administrative diagnosis.

^a ^Combined sample of suicide deaths and controls

^b ^Rounded to a whole number

^c ^The chart notation had to be clear it was referring to a suicide attempt occurring in the past 12 months.

### Alcohol Problem Drinking/Abuse/Dependence

Table 3 shows the administrative data vs. chart comparisons for alcohol, drug, and tobacco use diagnoses. Administrative codes for alcohol abuse or dependence had specificity greater than 96% in all three samples. Sensitivity, however, was lower than 74% in all three samples (weighted sensitivity estimates were lower than 78%), with the lowest sensitivity (53.8%) in the control sample. In the combined sample of cases and controls, sensitivity was 68.2%, specificity was 96.9%, positive predictive value of alcohol abuse diagnosis in administrative data was 87.4%, and negative predictive value was 90.8%.

**Table 3:** Alcohol dependence, illicit drug dependence and tobacco use diagnoses: comparison of administrative diagnoses against chart notation during the past 12 months prior to and including the index date

	*Alcohol Problems, Abuse/Dependence*				
	***Unweighted Estimates, %***^b ^***(N in fraction)***				
***Sample***	***Agreement ***	***Sensitivity***	***Specificity***	***PPV***	***NPV***

Suicide Death	90 (331/368)	74 (90/121)	98 (241/247)	94 (90/96)	89 (241/272)

Control	90 (327/362)	54 (28/52)	96 (299/310)	72 (28/39)	93 (299/323)

AD New User	90 (515/571)	63 (73/116)	97 (442/455)	85 (73/86)	91 (442/485)

Combined^a^	90 (658/730)	68 (118/173)	97 (540/557)	87 (118/135)	91 (540/595)

	***Weighted Estimates, %***^b ^***(95% confidence interval)***				

Suicide Death	90 (87, 94)	78 (70, 87)	97 (95, 100)	94 (88, 100)	89 (84, 93)

Control	90 (85, 95)	55 (34, 75)	97 (95, 99)	80 (67, 94)	91 (86, 96)

AD New User	91 (87, 94)	59 (46, 73)	98 (96, 100)	86 (75, 97)	91 (88, 95)

Combined^a^	90 (85, 95)	55 (35, 75)	97 (95, 99)	80 (68, 93)	91 (86, 96)

	***Illicit Drug Use/Abuse/Dependence***				

	***Unweighted Estimates, %***^b ^***(N in fraction)***				

	***Agreement ***	***Sensitivity***	***Specificity***	***PPV***	***NPV***

Suicide Death	90 (331/368)	74 (26/35)	92 (305/333)	48 (26/54)	97 (305/314)

Control	96 (348/362)	58 (11/19)	98 (337/343)	65 (11/17)	98 (337/345)

AD New User	93 (533/571)	70 (38/54)	96 (495/517)	63 (38/60)	97 (495/511)

Combined^a^	93 (679/730)	69 (37/54)	95 (642/676)	52 (37/71)	97 (642/659)

	***Weighted Estimates, %***^b ^***(95% confidence interval)***				

Suicide Death	90 (86, 94)	78 (62, 94)	92 (88, 95)	51 (35, 67)	97 (95 99)

Control	97 (94, 99)	61 (30, 91)	99 (97, 100)	70 (43, 96)	98 (96, 100)

AD New User	91 (85, 96)	54 (24, 85)	95 (92, 98)	58 (37, 79)	94 (89, 100)

Combined^a^	97 (94, 99)	61 (33, 89)	99 (97, 100)	69 (45, 93)	98 (96, 100)

	***Tobacco Use***				

	***Unweighted Estimates, %***^b ^***(N in fraction)***				

	***Agreement ***	***Sensitivity***	***Specificity***	***PPV***	***NPV***

Suicide Death	73 (267/368)	42 (70/167)	98 (197/201)	95 (70/74)	67 (197/294)

Control	76 (274/362)	36 (46/128)	97 (228/234)	89 (46/52)	74 (228/310)

AD New User	74 (424/571)	40 (95/237)	99 (329/334)	95 (95/100)	70 (329/471)

Combined^a^	74 (541/730)	39 (116/295)	98 (425/435)	92 (116/126)	70 (425/604)

	***Weighted Estimates, %***^b ^***(95% confidence interval)***				

Suicide Death	70 (64, 76)	42 (33, 52)	97 (94, 100)	94 (87, 100)	63 (56, 70)

Control	76 (70, 82)	39 (27, 50)	98 (96, 100)	91 (83, 99)	73 (66, 80)

AD New User	77 (72, 82)	44 (34, 54)	99 (97, 100)	95 (90, 100)	73 (67, 79)

Combined^a^	76 (70, 82)	39 (27, 50)	98 (96, 100)	91 (84, 99)	73 (66, 80)

Note: For alcohol dependence, kappa values are 0.76 for suicide cases, 0.57 for controls and 0.66 for AD new users. For illicit drug dependence, kappa values are 0.53 for suicide cases, 0.55 for controls and 0.63 for AD new users.

Abbreviation: NA is not available, AD is antidepressant; PPV is positive predictive value of an administrative diagnosis; NPV is the negative predictive value of an administrative diagnosis.

^a ^Combined sample of suicide deaths and controls

^b ^Rounded to a whole number

### Illicit Drug Problem Use/Abuse/Dependence

Specificity of administrative codes for chart notation of illicit drug use was higher than 90% across all samples (Table 3). Sensitivity, however, was much lower. The highest sensitivity (74.3%) was observed in the suicide death sample, and the lowest sensitivity (57.9%) was observed in the control sample. Positive predictive value of illicit drug use diagnosis in the administrative data was very low across the three samples, with the estimate in the combined suicide death and control samples of only 52.1% (weighted estimate was 69.5%).

### Tobacco Use

For tobacco use, the various accuracy measures varied least among the three different samples, and the unweighted and weighted estimates were similar. In the combined suicide death and control sample, sampling fraction-adjusted sensitivity was 38.6%, specificity 97.8%, positive predictive value of tobacco use diagnosis 91.2% and negative predictive value of tobacco use diagnosis 73.2%.

### By Age Group

For alcohol abuse/dependence, specificity remained very similar across the samples and between age groups (< 50 vs. ≥ 50 years old). On the other hand, sensitivity was higher in < 50 year olds than in ≥ 50 year olds in both the suicide and control samples. In the suicide sample, unweighted sensitivity was 85.2% (95% CI = 73.8-93.0%) in younger patients, and 63.3% (49.9-75.4%) in older patients, and similarly, in the control sample, sensitivity was 60.0% (38.7-78.9%) in younger patients and 48.1% (28.7-68.1%) in older patients. Prevalence of alcohol abuse/dependence based on chart data was about 2.2 times (p < 0.001) higher in the suicide than the control sample, and 1.8 times (p < 0.001) higher in the younger than the older subgroup within each sample. Total number of mental health visits over the prior 12 months of the index visits was also higher in the case sample than control sample, and higher in the younger than the older subgroup within each sample; mean number of mental health outpatient visits were 8.7, 4.6, 4.3 and 3.3 for younger subgroup in the suicide sample, older subgroup in the suicide sample, younger subgroup in the control sample, and older subgroup in the control sample, respectively.

### Estimated Prevalence across Data Sources

As an illustration of the potential impact of misclassification and differential misclassification, we calculated sampling fraction-adjusted prevalence of each condition based on chart data as well as administrative data (Table 4). In the combined suicide death and control sample, the prevalence of suicide attempt was 0.4% using chart data, while it was only 0.008% using administrative data. The prevalence of alcohol problem drinking/abuse/dependence was 17.6% vs. 12.0%, and of illicit drug problem use/abuse/dependence was 5.3% vs. 4.6%, using chart data vs. administrative data, respectively. Tobacco use diagnosis showed a bigger discrepancy than alcohol or drug dependence diagnoses where the prevalence was 36.8% using chart data, but only 15.6% using administrative data.

**Table 4:** Weighted prevalence estimates based on chart and on administrative (ICD-9) data

	*Suicide Attempt*		*Alcohol* *Abuse*		*Illicit Drug Abuse*		*Tobacco* *Use*	
***Sample***	***Chart***	***ICD-9***	***Chart***	***ICD-9***	***Chart***	***ICD-9***	***Chart***	***ICD-9***

Suicide Death	15.9	3.1	35.7	29.7	10.2	15.6	49.5	22.4

Control	0.3	0	17.5	11.9	5.3	4.6	36.8	15.5

AD New User	3.1	0.8	18.5	12.8	11.1	10.5	39.2	18.2

Suicides & Controls	0.4	0.008	17.6	12.0	5.3	4.6	36.8	15.6

## Discussion

Studies using administrative data rely on the accuracy of the ICD-9 diagnostic codes. This study was conducted to validate the administrative diagnoses of four key behavioural variables often used in mental health and health services research by comparing them to the presence of the corresponding conditions in chart notation.

Overall agreement and specificity were generally high across all behavioural variables. Sensitivity, however, was substantially lower than optimal (< 75%) for all four variables, and was particularly low for suicide attempt. Sensitivity was consistently highest in the suicide sample, followed by the antidepressant new user sample and lowest in the controls sample, likely due to more visits, service use and chart data for patients with greater severity or changes in severity. We note that due to the low prevalence of suicide attempts, the sensitivity estimates of suicide attempt are generally not as precise (e.g., one-sided 95% upper confidence limit for sensitivity of the control sample is 63%). Negative predictive values of administrative codes for behavioural variables were generally high, although positive predictive values varied. Positive predictive values of administrative codes for alcohol problems were 72-94%, and for tobacco use were 89-95%. However, for illicit drug use, PPV was only 48-65%.

Kashner *et al*. compared medical charts and administrative records for a random sample of 414 VHA inpatient discharges between July 1 and September 30, 1995 and found 93.7% agreement for alcohol dependence syndrome and 95.2% for drug dependence [13]. Our findings of 90.1% agreement for alcohol dependence and 96.1% agreement for drug dependence are similar to these findings. Their study did not report sensitivity and specificity; however, based on data presented in the paper, for alcohol dependence, their sensitivity and specificity were 69.4% and 95.5%, respectively. For illicit drug dependence, sensitivity and specificity were 72.1% and 96.6%, respectively. These findings of high specificity are similar to our results, but sensitivity is higher than that found in our study. This higher sensitivity in Kashner *et al*. may be due to basing the study on inpatient discharges rather than the more comprehensive data available from chart review. A more recent Canadian study based on 4,008 randomly selected patients admitted from January 1 to June 30, 2003 at four teaching hospitals in Alberta reported 53.6% sensitivity and 99.1% specificity for alcohol abuse and 55.3% sensitivity and 99.0% specificity for drug abuse by comparing ICD-9 based diagnoses against the chart diagnoses [14]. This finding is similar to ours, except we have slightly lower specificities (weighted accuracy of 97.2% for alcohol abuse and 98.5% for drug abuse).

The lower-than-desirable coding of these variables, and in particular of suicide attempt, might be anticipated. However, numerous studies have used these variables as covariates or even as primary endpoints [17]. Unfortunately, if misclassification is such that a large proportion of these behavioural variables (e.g., suicide attempts) are missed, it will lead to an under-estimation not only of the prevalence of the particular condition, but also may have an impact on effect size estimates of interest. In addition, when accuracy of classification is different across the different subgroups, the systematic bias often can mask an association or create a spurious one, depending on the study design. For example, if suicide attempt is more accurately identified in drug users than non-drug users, the differential accuracy of suicide attempt may potentially lead to a spurious association between drug use and suicide attempt. Increasing the sample size will not eliminate such biases.

Assuming that chart diagnosis is the gold standard, the generally high specificity means that over-estimation of the prevalence based on administrative data from false positives is not likely. On the other hand, the low sensitivity indicates that administrative data-based diagnoses are likely to under-estimate the prevalence, and this has been seen across all four behavioural diagnoses.

Although neither low sensitivity nor low specificity are desirable, the impact of drawing conclusions based on variables with low sensitivity combined with high specificity is likely less undesirable than the conclusions drawn from studies based on variables with low specificity and high sensitivity. In studies where variables with low specificity are used, false positives will likely bias the estimation of the effects of interest whether the variables are used as endpoints or as primary predictors. However, in studies where variables with low sensitivity are used as primary endpoints, mainly statistical power will be reduced due to under-identified events. Similarly, in studies where these variables are used as predictors or covariates, the predictive power will be compromised and thus any adjustments for selection bias, for example, will not be as effective.

There are limitations to this study. Our study used data from the 12 months prior to index date, and a greater number of visits or longer length of any inpatient stays within the 12 months are likely to give a greater amount of information in both charts and administrative databases. Thus our results do not necessarily generalize to level of agreement for a single visit or a single inpatient stay. Our results may not be fully generalizable to patients without a depression diagnosis or care delivered outside of the VHA or to care delivered during other time periods within the VA. We also note that the time period of this study precedes multiple clinical initiatives the VHA has taken to increase the detection of suicidal behaviour and reduce suicide risk. Clinical reminders requiring screening for tobacco use [18] is in the developmental stage in the VHA, and started nationally in 2008 for alcohol abuse/dependence (based on the AUDIT questionnaire) [19]. The VHA system potentially has fewer financial incentives to promote full diagnostic coding than many private sector settings, although the VHA allows up to 10 diagnostic fields for each encounter and has an electronic medical record that makes recording of conditions simple for busy clinicians, potentially enhancing the completeness of coding at each visit.

Another limitation is the lack of a true gold standard for these conditions. Both the chart notations and administrative diagnostic codes are limited to events that come to attention of VHA providers; thus medical records are a gold standard only in terms of recognized and diagnosed disorders that a clinician recorded. For substance use disorders, actual prevalence would, likely be much higher if validated diagnostic instruments were used. Many persons with such disorders are not identified and not treated. For instance, if a patient presents to an outside ER after a suicide attempt, this would not be captured within the VHA record unless they subsequently reported such an event to a medical or mental health provider. The goal of the study, however, is not to validate the administrative ICD-9 codes for suicide attempts and three substance use diagnoses using the true diagnosis, but to validate them using chart notation data which would be a more accurate, but more expensive source -- though less expensive than surveys -- of the behavioural disorder diagnoses in typical health services research studies.

Despite the limitations, the strength of our study is that it is based on samples drawn from complete nationwide records for all VHA patients, where all billing for patient care, even for specialists, occurs through the computer. We also note that our sampling was done to represent patients across region, years and gender and thus represent carefully the depression cohort at the VHA across regions over 5 years. Most importantly, to our knowledge this is the first study where agreement in suicide attempts determined by chart notation and E-codes was evaluated.

## Conclusions

Administrative data-based diagnoses among VHA records have high specificity but low sensitivity. However, the accuracy level varies by diagnosis and by patient subgroup. Given the lower than desirable level of accuracy, particularly of sensitivity, studies using behavioural diagnosis variables evaluated in this study as the primary endpoint or predictor should be careful in assessing the implication of potential misclassifications on their findings.

## Competing interests

The authors declare that they have no competing interests.

## Authors' contributions

HMK was involved in study design, statistical analysis and writing and editing of the manuscript. EGS participated in the writing. CMS participated in the design of the study, abstracted the charts and contributed to interpretation of data. DG participated in the study design, obtained the administrative data and was involved in editing of the manuscript. KZ participated in the study design and the editing of the manuscript. HW participated in the design of the study, abstracted the charts and contributed to interpretation of data. MV was involved in study design, and writing and editing of the manuscript. All authors read and approved the final manuscript.

## Pre-publication history

The pre-publication history for this paper can be accessed here:

http://www.biomedcentral.com/1472-6963/12/18/prepub

## Supplementary Material

Additional File 1 ***International Classification of Diseases, Ninth Revision, clinical modification *(*ICD-9-CM*) diagnoses codes used for determining presence of the administrative data based diagnoses**. Click here for file
