# Supplementary material for: Validation of key behaviourally based mental health diagnoses in administrative data: suicide attempt, alcohol abuse, illicit drug abuse and tobacco use
Source: BMC Health Serv Res. 2012 Jan 23;12:18. doi: 10.1186/1472-6963-12-18 (PMC3280157; doi:10.1186/1472-6963-12-18)
Supplement: Additional File 1 — International Classification of Diseases, Ninth Revision, clinical modification (ICD-9-CM) diagnoses codes used for determining presence of the administrative data based diagnoses. [file 1472-6963-12-18-S1.PDF]

Additional File 1: ***International Classification of Diseases, Ninth Revision, clinical modification*** **(*ICD-9*) diagnoses codes used for determining presence of the administrative data based diagnoses.**

Diagnoses	***Coded Diagnoses for Presence***	***Codes Denoting Absence*** [a]
Suicide Attempt	E950-E959	None
Alcohol Problem Drinking, Abuse or Dependence	303.0, 303.9, 305.0, 291.x	303.03, 303.93, 305.03
Other Drug Problem Use, Abuse or Dependence	304.2, 304.0, 304.7, 304.3, 304.1, 304.4, 304.5, 304.6,304.8,304.9, 305.6, 305.5, 305.2, 305.3, 305.4, 305.7, 305.8, 305.9, 292.x	304.23, 304.03, 304.73, 304.33, 304.13, 304.43, 304.53, 304.63, 304.83, 304.93, 305.63, 305.53, 305.23, 305.33, 305.43, 305.73, 305.83, or 305.93
Tobacco Use	305.1	None

[a] Absence of the codes or visits during the year were also coded as absence of the condition.
